# Supplementary material for: Zoledronic Acid as a Novel Dual Blocker of KIR6.1/2-SUR2 Subunits of ATP-Sensitive K+ Channels: Role in the Adverse Drug Reactions
Source: Pharmaceutics. 2021 Aug 27;13(9):1350. doi: 10.3390/pharmaceutics13091350 (PMC8465290; doi:10.3390/pharmaceutics13091350)
Supplement: Supplementary file 1 [file pharmaceutics-13-01350-s001.zip › pharmaceutics-1320039-supplementary.pdf]

pharmaceutics

Supplementary Materials: Zoledronic Acid as a Novel Dual Blocker of Kir6.1/2-Sur2 Subunits of ATP-Sensitive K^+ Channels: Role in the Adverse Drug Reactions

Maqoud Fatima, Scala Rosa, Tragni Vincenzo, Pierri Ciro Leonardo, Perrone Maria Grazia, Scilimati Antonio and Tricarico Domenico

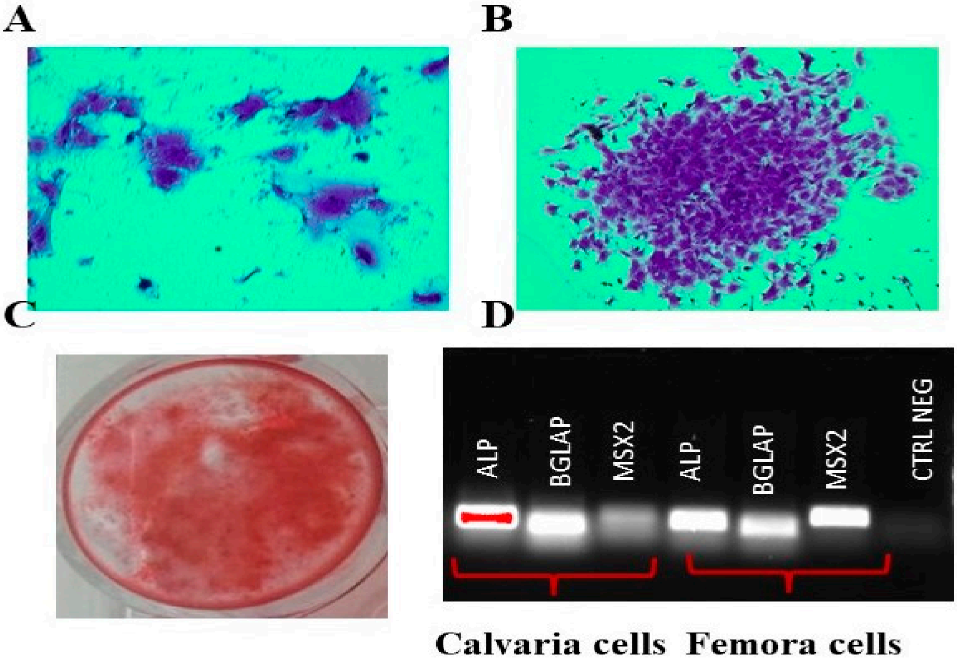

Figure S1. Primary bone cells are expected to be enriched in osteoblasts. Cells from femora (*A*) and calvaria chips (*B*) of WT/WT mice. Primary long bone cells can produce extracellular matrix, as revealed by Alizarin red staining, confirming the functional osteoblastic phenotype. (*C*) Femora and calvaria cell pellets were positive to Alkaline phosphatase (ALP), Osteocalcin (BGLAP), and Msh homeobox 2 **(**MSX2) genes in PCR analysis (*D*).

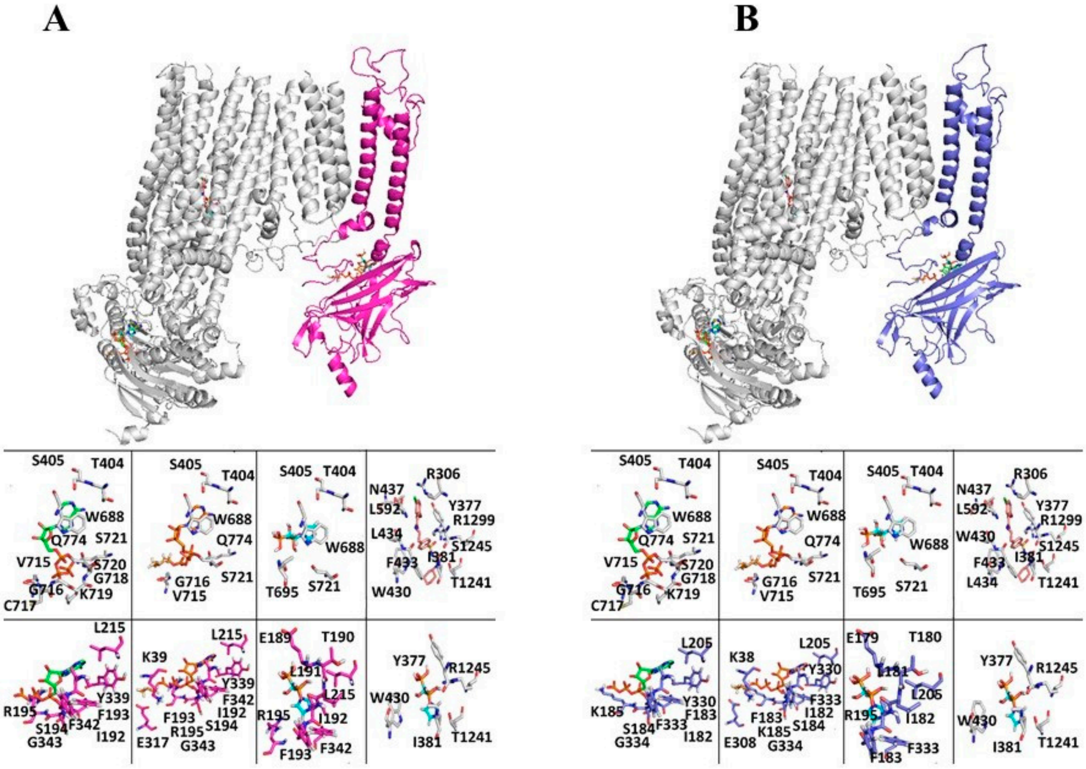

Figure S2. 3D comparative models of Kir.6.1-Sur1 human protein complex (**A**). Lateral views of Sur1 (grey cartoon)-Kir6.1 (magenta cartoon) human protein complex is reported in cartoon representation. In the panels below, within the first row, ATP (green sticks), ATP-IPP (orange sticks), zoledronic acid (cyan sticks) are reported in Sur1 ATP binding region (white sticks), whereas Glib (pink sticks) is reported in Sur1 – Glib binding region (in white sticks). In the panels below, within the second row, ATP (green sticks), ATP-IPP (orange sticks), zoledronic acid (cyan sticks) are reported in Kir.6.1 ATP binding region (magenta sticks). In addition, zoledronic acid is reported in the Sur1-Glib binding region (white sticks). (**B**) 3D comparative models of Kir.6.2-Sur1 human protein complex. Lateral views of Sur1 (grey cartoon)-Kir6.2 (dark blue cartoon) human protein complex is reported in cartoon representation. In the panels below, within the first row, ATP (green sticks), ATP-IPP (orange sticks), zoledronic acid (cyan sticks) are reported in Sur1 ATP binding region (white sticks), whereas Glib (pink sticks) is reported in Sur1 – Glib binding region (in white sticks). In the panels below, within the second row, ATP (green sticks), ATP-IPP (orange sticks), zoledronic acid (cyan sticks) are reported in Kir.6.2 ATP binding region (dark blue sticks). In addition, Zoledronic acid is reported in the Sur1-Glib binding region (white sticks).
